# Cytomegalovirus Viremia Predicts Postdischarge Mortality in Kenyan HIV-Exposed Uninfected Children^[Author-notes jiac047-FM1]^

**DOI:** 10.1093/infdis/jiac047

**Published:** 2022-03-11

**Authors:** Patricia B Pavlinac, Benson Singa, Meei-Li Huang, Lasata Shrestha, Vanessa Li, Hannah E Atlas, Mame Mareme Diakhate, Rebecca Brander, Liru Meshak, George Bogonko, Kirkby D Tickell, Christine J McGrath, Irine M Machuara, Derrick O Ounga, James A Berkley, Barbra A Richardson, Grace John-Stewart, Judd L Walson, Jennifer Slyker

**Affiliations:** Department of Global Health, University of Washington, Seattle, Washington, USA; Department of Epidemiology, University of Washington, Seattle, Washington, USA; Kenya Medical Research Institute, Nairobi, Kenya; Childhood Acute Illness and Nutrition Network, Nairobi, Kenya; Fred Hutchinson Cancer Research Center, Seattle, Washington, USA; Department of Laboratory Medicine, University of Washington, Seattle, Washington, USA; Department of Laboratory Medicine, University of Washington, Seattle, Washington, USA; Department of Global Health, University of Washington, Seattle, Washington, USA; Department of Global Health, University of Washington, Seattle, Washington, USA; Department of Global Health, University of Washington, Seattle, Washington, USA; Department of Global Health, University of Washington, Seattle, Washington, USA; Homa Bay Teaching and Referral Hospital, Homa Bay, Kenya; Kisii Teaching and Referral Hospital, Kisii, Kenya; Department of Global Health, University of Washington, Seattle, Washington, USA; Childhood Acute Illness and Nutrition Network, Nairobi, Kenya; Department of Global Health, University of Washington, Seattle, Washington, USA; Childhood Acute Illness and Nutrition Network, Nairobi, Kenya; Kenya Medical Research Institute, Nairobi, Kenya; Kenya Medical Research Institute, Nairobi, Kenya; Childhood Acute Illness and Nutrition Network, Nairobi, Kenya; Kenya Medical Research Institute-Wellcome Trust Research Programme, Kilifi, Kenya; Center for Tropical Medicine and Global Health, University of Oxford, Oxford, United Kingdom; Department of Global Health, University of Washington, Seattle, Washington, USA; Department of Biostatistics, University of Washington, Seattle, Washington, USA; Department of Global Health, University of Washington, Seattle, Washington, USA; Department of Epidemiology, University of Washington, Seattle, Washington, USA; Department of Medicine, Allergy, and Infectious Disease, University of Washington, Seattle, Washington, USA; Department of Pediatrics, University of Washington, Seattle, Washington, USA; Department of Global Health, University of Washington, Seattle, Washington, USA; Department of Epidemiology, University of Washington, Seattle, Washington, USA; Childhood Acute Illness and Nutrition Network, Nairobi, Kenya; Department of Medicine, Allergy, and Infectious Disease, University of Washington, Seattle, Washington, USA; Department of Pediatrics, University of Washington, Seattle, Washington, USA; Department of Global Health, University of Washington, Seattle, Washington, USA; Department of Epidemiology, University of Washington, Seattle, Washington, USA

**Keywords:** cytomegalovirus, HIV-1, child mortality, postdischarge mortality, azithromycin

## Abstract

**Background:**

Cytomegalovirus (CMV) viremia is associated with mortality in severely ill immunocompetent adults and hospitalized children with HIV (CWH). We measured CMV viremia in HIV-exposed and -unexposed Kenyan children aged 1–59 months discharged from hospital and determined its relationship with postdischarge mortality.

**Methods:**

CMV DNA levels were measured in plasma from 1024 children (97 of which were HIV exposed uninfected [HEU], and 15 CWH). Poisson and Cox proportional hazards regression models were used to identify correlates of CMV viremia ≥ 1000 IU/mL  and estimate associations with 6-month mortality, respectively.

**Results:**

CMV viremia was detected in 31% of children, with levels ≥ 1000 IU/mL in 5.8%. HIV infection, age < 2 years, breastfeeding, and midupper arm circumference < 12.5 cm were associated with CMV viremia ≥ 1000 IU/mL. Among HEU children, CMV ≥ 1000 IU/mL (hazard ratio [HR] = 32.0; 95% confidence interval [CI], 2.9–354.0; *P* = .005) and each 1-log increase in CMV viral load (HR = 5.04; 95% CI, 1.7–14.6; *P* = .003) were associated with increased risk of mortality. CMV viremia was not significantly associated with mortality in HIV-unexposed children.

**Conclusions:**

CMV levels at hospital postdischarge predict increased risk of 6-month mortality in Kenyan HEU children. CMV suppression may be a novel target to reduce mortality in HEU children.

**Clinical Trial Registration:**

NCT02414399.

## Teaser

Cytomegalovirus viremia was common in Kenyan children discharged from hospitals and high CMV viremia levels (*>*1000 IU/mL) were associated with 6-month mortality in HIV-exposed uninfected children. CMV suppression may be a novel target to reduce mortality in this high-risk population.

Mortality risks in the 6 months following hospital discharge are high for children in resource-limited settings globally (approximately 3%–18%) [1, 2], and are especially high in young children with malnutrition or human immunodeficiency virus (HIV) infection [2]. HIV-exposed uninfected children (HEU) also experience higher morbidity and mortality [3], and impaired growth compared to HIV-unexposed (HU) peers [4–6].

Increasing evidence suggests cytomegalovirus (CMV) may be an important contributor to poor outcomes in both immunocompetent and HIV-affected individuals admitted to hospital. Reactivation of CMV is common in immunocompetent adults admitted to intensive care units (ICU) and has been associated with higher risk of mortality, longer duration of hospitalization, increased risk of nosocomial infections, and greater oxygen dependency in multiple European- and US-based studies [7–11]. Primary CMV infection evokes a strong host inflammatory response, including increases in C-reactive protein, interleukin 6, and tumor necrosis factor-α [12–17]. The virus also has adapted several immune evasion strategies, including the production of host agonist and receptor mimics, which modulate host immune responses and may affect susceptibility to, or progression of, other infections [18]. Limited data suggest that prophylaxis with ganciclovir may improve oxygen use outcomes in septic adults with CMV viremia [19].

To date, no similar studies have been conducted in hospitalized immunocompetent children or adults in Africa; this is an important research gap given nearly universal CMV acquisition in early childhood, and high mortality rates in hospitalized African children [20–24]. Emerging data suggest CMV may have relevance for critically ill HIV-exposed children. In a cohort of hospitalized Kenyan children with HIV, the detection of CMV viremia at levels ≥ 1000 IU/mL at hospital admission was associated with a 74% increased risk of death or hospitalization at 15 days and longer hospital stay (5 days), independent of age and HIV RNA level [25].

The association between CMV viremia and outcomes among hospitalized HEU and HU children has not been previously reported. We investigated the association between CMV viremia at hospital discharge and 6-month mortality in children participating in a randomized controlled trial of azithromycin at hospital discharge [26].

## METHODS

### Enrollment and Follow-up of Study Participants

The Toto Bora trial (NCT02414399) recruited children between 1 and 59 months of age at discharge from 1 of 4 hospitals in western Kenya (Kisii Teaching and Referral Hospital, Homa Bay Teaching and Referral Hospital, St Paul Mission Hospital, and Kendu Adventist Mission Hospital) after admission for any conditions other than trauma, injury, or congenital abnormalities [26]. Children were randomized to receive placebo or azithromycin for 5 days (10 mg/kg on day 1, followed by 5 mg/kg on days 2–5), with the first dose being directly observed at the health facility. Children were followed for 6 months, with 2 scheduled follow-up visits (month 3 and month 6) where caregivers were interviewed about the child’s health history and a physical examination was performed. Anthropometric measurements were taken at all scheduled visits, as were blood samples collected in EDTA tubes, centrifuged, and resulting plasma and buffy coat stored at −80°C until further use. Height/length-for-age z-scores, weight-for-age z-scores, and weight-for-height/weight-for-length z-scores were calculated using the World Health Organization ANTHRO software [27]. Stunting and underweight were defined as height-for-age z-scores/length-for-age z-scores and weight-for-age z-scores < −2, respectively. Moderate wasting was defined as ≥ −3 to < −2 weight-for-height z-scores or ≥ 11.5 to < 12.5 cm midupper arm circumference, and severe wasting as weight-for-height z-scores < −3 or midupper arm circumference < 11.5 cm. Midupper arm circumference was only considered for wasting categorization in children 6 months or older. HIV status was determined by abstracting available data from medical records and/or testing during the hospitalization where indicated (per Kenyan Ministry of Health guidelines [28]). Dates of death and hospitalizations were determined through medical record abstraction (including nonrecruiting hospitals) and by self-report during scheduled or unscheduled visits. Cause of death was determined by clinical consensus after review of death certificates and medical records (when available), and verbal autopsies and assigned causes of death as described elsewhere [29].

### CMV Assays and Definitions

CMV DNA levels were measured from stored baseline plasma specimens using real-time quantitative polymerase chain reaction (PCR) as previously described; primers and probes are provided in Wamalwa et al [25]. The assay has a limit of detection of 1 copy/reaction, and values were transformed to express measurements in international units (IU)/mL by dividing by 1.4. We used a cutoff of 1000 IU/mL (high CMV viremia) as our primary outcome (for correlates analysis) and exposure (for mortality analysis) of interest, based on previous papers reporting an association with mortality in immunocompetent adults admitted to ICU [30], increased mortality and duration of hospitalization in hospitalized children with HIV (CWH) [25], and decreased lung function and stunting in Zimbabwean CWH [31].

### Statistical Analysis

Stata version 14 (StataCorp) was used for all analyses, and all comparisons were 2 sided with α = .05. Pearson χ^2^ test was used to compare CMV prevalence between groups of children based on HIV exposure, and the Wilcoxon rank-sum test was used to compare the distribution of CMV levels between groups. Correlates of CMV viremia were identified using Poisson regression to estimate prevalence ratios (PR) and 95% confidence intervals (CI), adjusting for baseline age ≥ 24 months, and HIV exposure or infection status. Cox proportional hazards regression was used to compare mortality rates associated with baseline CMV status (CMV viremia ≥ 1000 IU/mL and CMV viral load [log IU/mL]). Multivariable Cox regression models were adjusted for age. While randomization arm and site were considered as potential confounders, neither were retained in the model due to the lack of observed change in hazard ratios from an age-adjusted model by more than 10%. Two-way interaction terms between CMV and HIV exposure status were tested using a likelihood ratio test comparing models with and without (nested model) the interaction term in the model. Models and Kaplan-Meier curves were stratified by HIV exposure due to an a priori assumption that CMV viremia may be evoked and persist by unique mechanisms, and have differential consequences for HU, HEU, and CWH. Also, in secondary analyses we evaluated the association between CMV and mortality in groups of children defined by stunting and wasting status (among HU children).

The study was approved by the institutional review boards at the Kenya Medical Research Institute, the Kenya Pharmacy and Poisons Board, and the University of Washington. Caregivers provided informed written consent (or oral consent with a witnessed thumbprint) in their preferred local language (English, Kiswahili, Kisii, or Dholuo).

## RESULTS

### Participant Characteristics

The first 1024 of the 1400 enrolled Toto Bora participants (73%) had CMV PCR testing performed and were included in this analysis; characteristics are provided in Table 1. The median duration of hospital stay among the 1024 children included in this analysis was 3 days, with 17.6% admitted for 7 days or longer. A high proportion of children were stunted (23.9%), and many children were underweight (12.4%) or wasted (9.0%). In total, 118 (11.5%) of enrolled children had documented HIV exposure or infection; 103 (10.1%) were HEU and 15 (1.5%) had HIV infection (CWH). There were 6 children who were HIV exposed but infection status unknown and 34 children confirmed to be HIV uninfected, but in whom HIV-exposure status was unknown.

**Table 1.** Characteristics of Participants Who Were Tested for CMV Viremia at Hospital Discharge

Characteristic	Overall (n = 1024^a^)	CWH (n = 15)	HEU Children (n = 97)	HU Children (n = 872)
Site				
Kisii Teaching and Referral Hospital	628 (61.3)	9 (60.0)	21 (21.7)	575 (65.9)
Homa Bay County Referral Hospital	385 (37.6)	6 (40.0)	76 (78.4)	286 (32.8)
St Paul Mission Hospital	11 (1.1)	0 (0)	0 (0)	11 (1.3)
Sociodemographic				
Female	402 (39.3)	9 (60.0)	38 (39.2)	336 (38.6)
Age at enrollment, mo				
Median (IQR)	18 (9–32)	30 (16–38)	22 (12–36)	17.5 (9–31)
<12	336 (32.8)	3 (20.0)	24 (24.7)	295 (33.8)
12–23	306 (29.9)	4 (26.7)	29 (29.9)	265 (30.4)
24–60	382 (37.3)	8 (53.3)	44 (45.4)	312 (35.8)
Duration of hospital admission, d, median (IQR), n = 1015	3 (2–5)	7 (3–10)	4 (2–6)	3 (2–5)
Hospital stay > 7 d, n = 1015	179 (17.6)	8 (53.3)	19 (19.8)	140 (16.2)
Nutritional status				
Stunted, n = 1020	244 (23.9)	7 (46.7)	33 (34.0)	188 (21.6)
Underweight, n = 1023	127 (12.4)	3 (20.0)	15 (15.5)	95 (10.9)
Wasting, n = 1024	92 (8.98)			
Moderate^b^	56 (5.47)	2 (13.3)	4 (4.1)	47 (5.4)
Severe^c^	36 (3.52)	2 (13.3)	6 (6.2)	22 (2.5)
Midupper arm circumference < 12.5 cm among > 6 mo olds, n = 887	66 (7.44)	5 (33.3)	13 (13.4)	99 (11.4)
Breastfeeding				
During hospitalization	503 (49.1)	4 (26.7)	26 (27.1)	463 (53.3)
Among < 24 mo olds, n = 640	483 (75.5)	4 (57.1)	25 (47.2)	444 (79.6)
HIV status				
Unexposed^d^	872 (88.6)	…	…	…
HIV exposed^e^	118 (11.9)	…	…	…
HIV exposed uninfected	97 (10.1)	…	…	…
HIV infected	15 (1.46)	…	…	…

Data are No. (%) except where indicated.

Abbreviations: CMV, cytomegalovirus; CWH, children with HIV; HEU, HIV exposed uninfected; HIV, human immunodeficiency virus; HU, HIV unexposed; IQR, interquartile range.

Overall sample size includes 34 children whose HIV exposure status was unknown and 6 HIV-exposed children with unknown HIV-infection status.

Defined as ≥ −3 to < −2 weight-for-height z-score or ≥ 11.5 to< 12.5 cm midupper arm circumference in children 6 months or older.

Defined as weight-for-height z-score < −3 or mid upper arm circumference < 11.5 cm in children 6 months or older.

Excludes 34 child with unknown HIV exposure status.

Includes 6 HIV-exposed children with unknown HIV-infection status.

### CMV Detection and Levels

**Figure 1.**
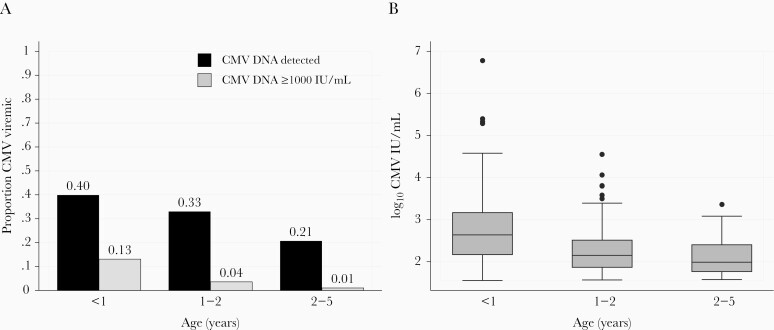
Cytomegalovirus (CMV) detection and levels in Kenyan infants, toddlers, and children at hospital discharge. *A*, Proportion of children with CMV detection above the limit of detection (black bars, *P* < .001 for comparison across ages) and ≥ 1000 IU/mL (gray bars, *P* < .001 for comparison across ages). *B*, Boxes show CMV viral load medians and interquartile ranges among children with detectable viremia at hospital discharge. Whiskers show upper adjacent values and outside values. *P* < .0001 for younger than 1 year versus 1–2 year olds; *P* = .07 for 1–2 versus 2–5 year olds.

All 1024 participants with CMV PCR testing had valid results at enrollment. At hospital discharge, 314 children had detectable CMV viremia (31%), with 5.8% having CMV levels ≥ 1000 IU/mL (Table 2). Infants < 1 year old had the highest prevalence of CMV detection and the highest median level of CMV viremia, with children 1–2 years and > 2 years of age having successively lower prevalence of viremia and CMV viral loads (Figure 1). The prevalence of CMV viremia and the median CMV viral load was similar between HEU and HU children (*P* > .05 for each comparison). However, 6 (0.69%) HU had very high CMV levels (≥10 000 IU/mL); 2 HU children, 5 and 7 months of age, had CMV levels ≥ 100 000 IU/mL and 4 children, between the ages of 5 and 13 months, had levels 10 000–100 000 IU/mL. Among HEU children, 3 (2.9%) had CMV levels 10 000–100 000 IU/mL and none were ≥ 100 000 IU/mL. Among CWH, 2 had CMV viral loads ≥ 100 000 IU/mL. While the total number of CWH in the cohort was small (n = 15), these children had significantly higher median CMV viral loads (3.2 log_10_ IU/mL; interquartile range [IQR], 2.6–5.3) and there was a higher proportion of children with viral loads ≥ 1000 IU/mL (20.0%) as compared to the HU children (median, 2.3 log_10_ IU/mL; IQR, 1.9–2.7 and 5.2% ≥ 1000 IU/mL; *P* = .012 and *P* = .015, respectively).

**Table 2.** Plasma CMV DNA Detection and Levels in HIV Exposed and Unexposed Kenyan Children at Hospital Discharge

HIV Status	Total No.	CMV Viremia, No. (%), *P* Value	CMV Viremia *≥* 1000 IU/mL, No. (%), *P* Value	log_10_ CMV IU/mL in Viremics, Median (IQR), *P* Value
All children	1024	314 (30.7)	59 (5.8)	2.3 (1.9–2.7)
HIV unexposed	872	253 (29.0)	45 (5.2)	2.3 (1.9–2.7)
HIV exposed^a^	118	45 (38.1), *P* = .061^b^	10 (8.5), *P* = .18^c^	2.4 (2.0–2.9), *P* = .24^d^
HIV exposed uninfected	97	36 (37.1), *P* = .088^b^	6 (6.2), *P* = .56^c^	2.4 (1.9–2.7), *P* = .74^d^
HIV infected	15	6 (40.0), *P* = .39^b^	3 (20.0), *P* = .015^c^	3.2 (2.6–5.3), *P* = .012^d^

Abbreviations: CMV, cytomegalovirus; HIV, human immunodeficiency virus; IQR, interquartile range.

HIV-exposed or infected group includes 6 children who were HIV exposed but whose HIV infection status was unknown.

Comparison to CMV detection in HIV-unexposed children, χ^2^ test.

Comparison to CMV detection ≥ 1000 IU/mL in HIV-unexposed children, χ^2^ test.

Comparison of CMV viral load distribution to HIV-unexposed children, Wilcoxon rank-sum test.

No single diagnosis explained the high frequency of CMV viremia in this study population. Children admitted with sepsis had the highest median CMV viral load at hospital discharge (2.69 log_10_ IU/mL) and 3.3% of children with sepsis had ≥ 1000 IU/mL (Table 3). Children diagnosed with pneumonia (7.3%), diarrhea (6.4%), and anemia (6.8%) were the most likely to have viral loads ≥ 1000 IU/mL.

**Table 3.** Prevalence and Levels of CMV Viremia at Hospital Discharge, by Diagnosis^a^

Diagnosis	No. of Cases	CMV Viremia, No. (%)	CMV ≥ 1000 IU/mL, No. (%)	log_10_ CMV IU/mL in Viremics, Median (IQR)
Pneumonia	314	108 (34)	23 (7.3)	2.41 (1.91–2.78)
Malaria	278	86 (31)	11 (4.0)	2.20 (1.85–2.58)
Diarrhea	188	60 (32)	12 (6.4)	2.28 (1.84–2.89)
Anemia	133	48 (36)	9 (6.8)	2.36 (1.92–2.67)
Sickle cell^b^	70	17 (24)	1 (1.4)	1.88 (1.70–2.12)
Suspected meningitis	53	12 (23)	2 (3.8)	2.43 (2.01–2.86)
Sepsis	30	11 (37)	1 (3.3)	2.69 (2.04–2.88)
Tuberculosis	15	2 (13)	0	1.92 (1.70–2.15)

Abbreviations: CMV, cytomegalovirus; IQR, interquartile range.

Some children had multiple diagnoses and appear in more than 1 category.

Includes sickle cell crisis as well as sickle cell disease as these were not always distinguished in medical record.

### Correlates of CMV Viremia

Baseline demographics, nutritional status, and HIV exposure were assessed as correlates of CMV viremia ≥ 1000 IU/mL at hospital discharge (Table 4). Effect sizes for each covariate were similar for HU and HEU children in crude and adjusted models (data not shown), so data are presented for the cohort overall. When adjusting for HIV exposure, children < 24 months were over 8 times more likely to have CMV viremia ≥ 1000 IU/mL at hospital discharge than older children (age-adjusted PR [aPR], 8.27; 95% CI, 3.03–23.1; *P* < .001). HIV infection was also highly associated with CMV viremia (aPR, 4.98; 95% CI, 1.55–16.1; *P* = .007), but HEU status was not significantly associated with a greater prevalence of viremia (aPR, 1.36; 95% CI, .58–3.20; *P* = .5). Adjusting for age and HIV exposure, caregiver-reported breastfeeding during the hospital stay, among those younger than 24 months of age, was the only other predictor of CMV viremia (aPR, 2.26; 95% CI, 1.05–4.87; *P* = .04).

**Table 4.** Correlates of CMV DNA *>* 1000 IU/mL at Hospital Discharge in Kenyan Children

Characteristic	CMV DNA ≥ 1000 IU/mL Prevalence in Correlate Group, No. (%)	Crude Prevalence Ratio (95% CI), *P* Value	Age and HIV-Exposure-Adjusted Prevalence Ratio (95% CI), *P* Value
Male	32 (5.2)	Ref	Ref
Female	27 (6.8)	1.31 (.785–2.2), *P* = .3	1.35 (.808–2.25), *P* = .3
Age > 24 mo	4 (1.1)	Ref	Ref
Age < 24 mo	55 (8.6)	8.18 (2.97–22.6), *P* < .001	8.27 (3.03–23.1), *P* < .001
Not underweight	48 (5.4)	Ref	Ref
Underweight	11 (8.7)	1.62 (.840–3.11), *P* = .2	1.30 (.672–2.51), *P* = .4
Not wasted	51 (5.5)	Ref	Ref
Moderate wasting	4 (7.1)	1.31 (.472–3.61), *P* = .6	1.19 (.430–3.30), *P* = .7
Severe wasting	4 (11.1)	2.03 (.734–5.62), *P* = .2	1.31 (.470–3.66), *P* = .6
Not stunted	49 (6.3)	Ref	Ref
Stunted	10 (4.1)	0.649 (.329–1.28), *P* = .2	0.660 (.332–1.31), *P* = .2
Midupper arm circumference > 12.5 cm	28 (3.4)	Ref	Ref
Midupper arm circumference < 12.5 cm	6 (9.1)	2.68 (1.10–6.44), *P* = .03^a^	1.88 (.769–4.60), *P* = .2^a^
In hospital < 7 days	45 (5.4)	Ref	Ref
In hospital *>* 7 days	13 (7.3)	1.35 (.728–2.50), *P* = .3	1.19 (.641–2.22), *P* = .6
Not breastfed while in hospital	7 (4.5)	Ref	Ref
Breastfed while in hospital	47 (9.7)	1.92 (.908–4.06), *P* = .09^b^	2.26 (1.05–4.87), *P* = .04^b^
HIV unexposed	45 (5.2)	Ref	Ref
HIV exposed uninfected	6 (6.2)	1.20 (.511–2.81), *P* = .7	1.36 (.582–3.20), *P* = .5^c^
HIV infected	3 (20.0)	3.88 (1.15–11.9), *P* = .02	4.98 (1.55–16.1), *P* = .007^c^

Adjusted for age *>* 24 months and HIV exposure (HIV exposed uninfected or infected).

Abbreviations: CI, confidence interval; CMV, cytomegalovirus; HIV, human immunodeficiency virus; PR, prevalence ratio.

Midupper arm circumference point estimate among children > 6 months old (n = 887).

Breastfeeding point estimate among children < 24 months old (n = 640), adjusted only for HIV exposure.

Adjusted only for age *>* 24 months.

### CMV Viremia and Risk of Mortality After Hospital Discharge

**Figure 2.**
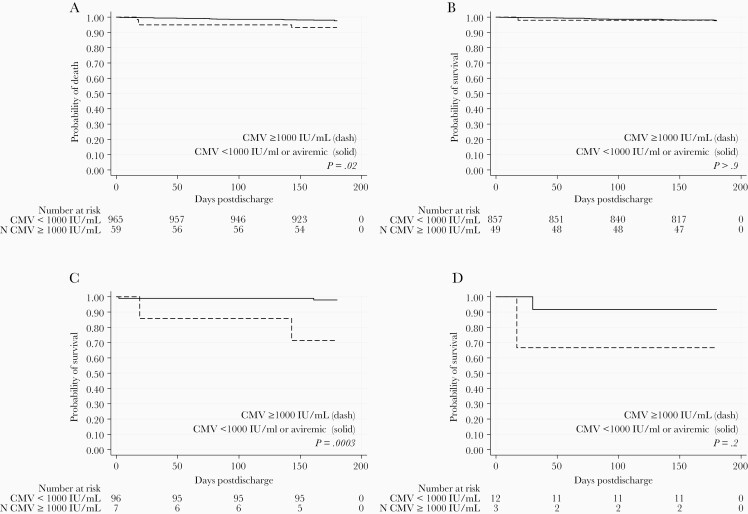
Six-month postdischarge mortality in cytomegalovirus (CMV) viremic and aviremic children stratified by HIV exposure. Kaplan-Meier functions are shown for children grouped by CMV viremia and HIV exposure status. *P* values were determined by log-rank test for equality of survivor function. CMV viremia was categorized as a binary variable ≥ 1000 IU/mL (dashed line) versus aviremic or < 1000 IU/mL (solid line) for (*A*) all children, n = 1024, (*B*) HIV-unexposed children, n = 906, (*C*) HIV-exposed uninfected children, n = 103, and (*D*) children with HIV, n = 15.

Overall, there were 24 deaths among the 1024 included children during the 6 months of follow-up (2.3%). Thirteen deaths were due to infectious causes (pneumonia [n = 2], malaria [n = 1], and  diarrhea [n = 1], central nervous system infections [n = 2], and unknown cause of death with signs of infection [n = 7]) and 11 from noninfectious causes (congestive heart failures [n = 2], anemia [n = 3], sickle cell crisis [n = 1], and unknown without signs of infection [n = 5]). Figure 2 shows the Kaplan-Meier curves for children overall and grouped by HIV exposure and infection status. Survival rates in children with low-level CMV viremia (<1000 IU/mL) were similar to those of children without detectable CMV viremia (*P* = .70). However, children with high-level CMV viremia (*≥*1000 IU/mL) had lower survival rates when compared both to children without detectable CMV (*P* = .02) and to children with low-level viremia <1000 IU/mL (*P* = .08; Supplemental Figure 1). As a result, in subsequent analyses we compared mortality rates between children with high-level (*≥*1000 IU/mL) versus low-level or no viremia (Figure 2).

Children with CMV level ≥ 1000 IU/mL at hospital discharge were close to 3 times more likely to die than children with low-level or no viremia adjusting for age (aHR, 2.78; 95% CI, .91–8.50), albeit at borderline statistical significance (*P* = .07; Table 5). Stratification by HIV exposure revealed that this association was driven by HIV-exposed children (likelihood ratio test of interaction between HIV-exposure status and CMV viral load > 1000 IU/mL *P* = .048, and CMV log_10_ IU/mL level *P* = .011). In HU children, neither CMV viral load ≥ 1000 IU/mL (aHR, 1.16; 95% CI, .15–8.8; *P* = .89) nor CMV log_10_ IU/mL levels (aHR, 1.05; 95% CI, .47–2.32; *P* = .91) were associated with risk of death in crude or adjusted models. While there were 14 deaths among HU children, there was only a single death among HU children with CMV viral load > 1000 IU/mL. Further stratification in the HU group by malnutrition status did not reveal evidence of an association between CMV and death (Supplementary Table 2). HEU children with CMV level ≥ 1000 IU/mL had a much higher risk of death than children with low-level or no viremia (aHR, 32.0; 95% CI, 2.90–353.95; *P* = .005), and each 1-log increase in CMV IU/mL was associated with a 5-fold increased risk of death (aHR, 5.04; 95% CI, 1.74–14.60; *P* = .003). There were only 2 deaths observed among the 15 CWH, 1 in each CMV group, limiting the statistical power in this subgroup analysis. However, the magnitude of age-adjusted hazard ratios were similar to those observed in HEU children (CMV viral load ≥ 1000 IU/mL aHR, 10.7; 95% CI, .04–3056.00; *P* = .41, and 1-log increase in CMV IU/mL aHR, 2.58; 95% CI, .692–9.64; *P* = .26).

**Table 5.** Incidence Rates and Hazard Ratios for Mortality by CMV Viremia at Hospital Discharge

CMV Viremia	n	Deaths, No.	Person-Years	IR (95% CI) per 100 Person-Years	HR (95% CI), *P* Value	Age-Adjusted HR (95% CI),^a^*P* Value
All infants (n = 1024)						
CMV ≥ 1000 IU/mL or aviremic	965	20	445	4.5 (2.9–7.0)	Ref	Ref
CMV ≥ 1000 IU/mL	59	4	26.3	15.2 (5.7–40.6)	3.4 (1.2–9.9), *P* = .03	2.8 (.9–8.5), *P* = .07
CMV level in log_10_ IU/mL	1024	24			2.0 (1.4–2.9) –*P* < .001	1.9 (1.2–2.8), *P* = .003
HU children (n = 872)						
CMV ≥ 1000 IU/mL or aviremic	827	16	395	4.2 (2.56–6.8)	Ref	Ref
CMV ≥ 1000 IU/mL	45	1	22.5	4.8 (.7–34.3)	1.2 (.2–8.8), *P* = .89	0.9 (.1–6.7), *P* = .90
CMV level in log_10_ IU/mL	872	17			1.1 (.5–2.3), *P* = .91	0.9 (.4–2.0), *P* = .788
HEU children (n = 97)						
CMV ≥ 1000 IU/mL or aviremic	91	2	42.4	2.36 (.33–16.75)	Ref	Ref
CMV ≥ 1000 IU/mL	6	2	2.3	86.25 (21.57–344.85)	32.0 (2.9–354.0), *P* = .005	195.7 (1.3–28654.2), *P* = .038
CMV level in log_10_ IU/mL	97	4			5.04 (1.7–14.6), *P* = .003	9.84 (1.8–54.3), *P* = .009
CWH (n = 15)						
CMV ≥ 1000 IU/mL or aviremic	12	1	5.3	18.8 (2.7–133.4)	Ref	Ref
CMV ≥ 1000 IU/mL	3	1	1.0	100.1 (14.1–710.4)	4.90 (.3–79.5), *P* = .26	10.74 (.04–3056.0), *P* = .41
CMV level in log_10_ IU/mL	15	2			1.8 (.87–3.77), *P* = .11	2.6 (.692–9.64), *P* = .16

Abbreviations: CI, confidence interval; CMV, cytomegalovirus; CWH, children with HIV; HEU, HIV exposed uninfected; HIV, human immunodeficiency virus; HR, hazard ratio; HU, HIV unexposed; IR, incidence rate.

Site and randomization were not retained in the adjusted model because they did not meaningfully change the point estimates.

## DISCUSSION

In this cohort of Kenyan children assessed at hospital discharge, we found that nearly a third had detectable CMV viremia in their blood, with some children having extremely high CMV viral loads. Infants (aged ≤12 months), those living with HIV, and who were currently breastfeeding had the highest prevalence of CMV viremia ≥ 1000 IU/mL. Independent of age, the detection of CMV at ≥ 1000 IU/mL was associated with an increased risk of mortality over the next 6 months in HEU children, as was each 1-log_10_ increase in CMV viral load.

To the best of our knowledge, this is the first study to assess the association between CMV viremia at hospital discharge and mortality. Our data suggest that CMV viremia ≥ 1000 IU/mL at hospital discharge is strongly associated with risk of mortality in HEU children, with each 1-log increase in CMV viremia associated with a 5-fold increased risk of death. HEU children experience double the risk of hospitalization and death than their HU counterparts [32, 33], yet the mechanism underpinning this vulnerability are not fully understood. The intersecting and complex sociodemographic, behavioral, and physiologic factors, including maternal disease experience and antiretroviral therapy exposure, altered immune ontogeny during fetal development, and exposure to more maternal infections likely all play a role [34]. All of these factors could alter CMV pathogenesis in HEU children, and this assumption is supported by previous work demonstrating high-level and prolonged CMV viremia in otherwise healthy HEU infants during primary CMV infection [35–37].

Given prior reported associations between CMV reactivation and mortality in severely ill hospitalized adults [7–11] and CWH [25], these findings suggest that CMV viremia may also be an important marker of risk in HEU children. This is especially remarkable given the potential for survival bias among children enrolled at hospital discharge, as many of the sickest children may not have survived to enrollment. These findings are also consistent with previous data published from our group demonstrating that among a large cohort of CWH admitted to hospital, CMV viremia ≥ 1000 IU/mL was associated with a 74% increased risk of death or continued hospitalization at 15 days and with 5 days longer hospitalization [25]. While we did not find an association between CMV viremia and mortality in HU children, we caution that the study had limited statistical power to detect more modest, but still important, associations with mortality in this subgroup. Notably, survival bias excludes the sickest children from our study; if we had enrolled at hospital admission, we may have found an association between CMV and inpatient mortality and more research is needed to determine if hospitalized HU children should be included in CMV-targeted interventions.

We are unable to determine whether CMV viremia is causally linked to mortality, or if it is simply a marker of more severe illness, a barometer of immune competence, or an unidentified cofactor. A number of children in our study had extremely high CMV viral loads; while CMV was not a specific diagnosis in any participant included in this study, it is possible that children with the highest CMV viral loads may have had CMV disease and not just viremia. CMV has a wide tissue tropism, and can replicate in the lung, central nervous system, and gut, causing pneumonitis, neurologic diseases, enteritis, and esophagitis [38–41]. To date, limited trial data exist to support or refute a causal role for CMV. In a randomized control trial of 156 mechanically ventilated CMV-seropositive adults with trauma or sepsis, randomization to the antiviral drug ganciclovir increased the number of ventilator-free days, and decreased the duration of mechanical ventilation compared to placebo; notably, these benefits were observed only in those with sepsis [19].

The prevalence of CMV viremia in all children, regardless of HIV exposure history, was high and was in the range of CMV reactivation rates (5%–50%) found in studies of immunocompetent, severely ill adults admitted to ICU [7, 8]. CMV viremia was associated with younger age and currently breastfeeding, although detectable virus was also found in a substantial proportion of children older than 2 years (21%). In our previous birth cohort studies, we found that virtually all HIV-exposed children acquired CMV infection before the first year of life, and that high-level and prolonged CMV viremia (>3 months in duration) was common, with most CWH and HEU having detectable CMV viremia at least 6 months, and some continuing to be viremic for more than a year [35–37].

Strengths of the study include the well-characterized and large study population, complete ascertainment of consecutive enrollment samples, and inclusion of a large number of HEU and HU children. However, the total number of deaths was small and the effect sizes reported here have limited precision. With only 15 CWH included, we were underpowered to confirm an association between CMV viremia and postdischarge mortality in this population. It was also not possible to verify HIV infection status in 6 children, nor to capture any new HIV infections postdischarge, and HIV treatment and laboratory measures (CD4, HIV RNA levels) were not available in the CWH. Admission diagnoses were abstracted from medical records and may be subject to misclassification. As noted above, assessment of children at hospital discharge, rather than admission, biases inclusion for children with less-severe disease, and could reduce observed associations between CMV viremia and mortality. Finally, because we did not perform CMV serology, we cannot determine CMV infection status in the CMV aviremic children.

In summary, these data suggest that CMV viremia is common in hospitalized Kenyan children and that in HEU children CMV viremia ≥ 1000 IU/mL identifies a subset of children at high risk for death. These data support the need for interventional studies to be conducted in hospitalized, African CWH and HEU who have a high risk for both inpatient and postdischarge mortality, as well as more research into mechanisms of CMV reactivation, persistence, and pathogenesis in the inpatient setting.

## Supplementary Data

Supplementary materials are available at *The Journal of Infectious Diseases* online. Supplementary materials consist of data provided by the author that are published to benefit the reader. The posted materials are not copyedited. The contents of all supplementary data are the sole responsibility of the authors. Questions or messages regarding errors should be addressed to the author.

jiac047_suppl_Supplementary_Figure_S1

jiac047_suppl_Supplementary_Table_S1

jiac047_suppl_Supplementary_Table_S2

jiac047_suppl_Supplementary_Data

## Data Availability

The data that support the findings of this study are available from the corresponding author upon reasonable request.

## Notes

***Acknowledgment***. We are grateful to Bhavna Chohan and Brian Khasimwa who provided training in plasma collection.

***Financial support.*** This work was supported by the Eunice Kennedy Shriver National Institute of Child Health and Human Development, National Institutes of Health (grant numbers R01 HD079695 and R01 HD079695-S to J. W.); the University of Washington Center for AIDS Research (grant number P30 AI027757); and the University of Washington Global Center for Integrated Health of Women, Adolescents, and Children.
