# Supplementary material for: Cytomegalovirus Viremia Predicts Postdischarge Mortality in Kenyan HIV-Exposed Uninfected Children
Source: J Infect Dis. Author manuscript; Available in PMC 2022 Nov 8. (PMC9624454; doi:10.1093/infdis/jiac047)
Supplement: Supp. 1 [file EMS152549-supplement-Supp__1.docx]

| **Supplemental Table 1. Median discharge CMV viral loads in survivors and mortalities.** | | | |
| --- | --- | --- | --- |
|  | **N** | **Median CMV log_10_**  **IU/ml (IQR)** | **P** |
| **All children** | **1024** |  |  |
| Survivors | 1000 | 1.40 [1.40, 1.79] | 0.08 |
| Deaths | 24 | 1.40 [1.40, 2.46] |  |
| **HU children** | **906** |  |  |
| Survivors | 888 | 1.40 [1.40, 1.76] | 0.9 |
| Deaths | 18 | 1.40 [1.40, 2.01] |  |
| **HEU children** | **103** |  |  |
| Survivors | 99 | 1.40 [1.40, 1.94] | 0.0005 |
| Deaths | 4 | 3.41 [2.70, 4.05] |  |
| **CWH** | **15** |  |  |
| Survivors | 13 | 1.40 [1.40, 2.58] | 0.4 |
| Deaths | 2 | 4.09 [1.40, 6.78] |  |

**Notes.** P value from Mann-Whitney U test.
