# Supplementary material for: Cytomegalovirus Viremia Predicts Postdischarge Mortality in Kenyan HIV-Exposed Uninfected Children
Source: J Infect Dis. Author manuscript; Available in PMC 2022 Nov 8. (PMC9624454; doi:10.1093/infdis/jiac047)
Supplement: Supp. 2 [file EMS152549-supplement-Supp__2.docx]

| **Supplemental Table 2. Incidence rates and hazard ratios for mortality by CMV viremia at hospital discharge overall and by wasting and stunting status among those HIV unexposed children** | | | | | | |
| --- | --- | --- | --- | --- | --- | --- |
|  | **N** | **Deaths** | **Person-years** | **IR [95%CI]**  **per 100 PY** | **HR [95%CI],**  **p value** | **Age-adjusted HR [95%CI]^a^, p value** |
| **Non-wasted children (n=829)** |  |  |  |  |  |  |
| CMV <1000 or aviremic | 774 | 13 | 364.0 | 3.6 [2.1, 6.2] | [ref] | [ref] |
| CMV >1000 IU/ml | 41 | 1 | 19.3 | 5.2 [0.7, 36.8] | 1.5 [0.2, 11.2], p=0.72 | 0.9 [0.1, 7.2], p=0.94 |
| CMV level in log_10_ IU/ml | 815 | 14 |  |  | 1.2 [0.6, 2.7], p=0.59 | 1.0 [0.4, 2.2], p=0.97 |
| **Wasted children (n=77)** | | | | | | |
| CMV <1000 or aviremic | 66 | 4 | 31.1 | 12.9 [4.8, 34.3] | [ref] | [ref] |
| CMV >1000 IU/ml | 7 | 0 | 3.2 | 0 | Not estimable | Not estimable |
| CMV level in log_10_ IU/ml | 97 | 4 |  |  | Not estimable | Not estimable |
| **Non-stunted children (n=700)** | | | | | | |
| CMV <1000 or aviremic | 647 | 12 | 304.5 | 3.9 [2.2, 6.9] | [ref] | [ref] |
| CMV >1000 IU/ml | 41 | 0 | 19.2 | 0 | Not estimable | Not estimable |
| CMV level in log_10_ IU/ml | 688 | 12 |  |  | 0.93 [0.3, 2.5], p=0.89 | Not estimable |
| **Stunted children (n=202)** | | | |  |  |  |
| CMV <1000 or aviremic | 189 | 5 | 88.7 | 5.6 [2.3, 13.5] | [ref] | [ref] |
| CMV >1000 IU/ml | 7 | 1 | 3.3 | 30.3 [4.3, 215.4] | 5.2 [0.6, 44.6], p=0.13 | 0.9 [0.3, 2.5], p=0.83 |
| CMV level in log_10_ IU/ml | 196 | 6 |  |  | 1.2 [0.3, 4.7], p<0.81 | 0.7 [0.2, 2.9], p=0.64 |
