# Supplementary material for: Cytomegalovirus Viremia Predicts Postdischarge Mortality in Kenyan HIV-Exposed Uninfected Children
Source: J Infect Dis. 2022 Mar 11;226(9):1519–27. doi: 10.1093/infdis/jiac047 (PMC9624454; doi:10.1093/infdis/jiac047)
Supplement: Supplementary file 1 [file jiac047_suppl_supplementary_data.docx]

**Supplemental Figure 1. Six-month post-discharge mortality rates in CMV viremic, low viremic, and high-viremic children**. Kaplan-Meier functions are shown for children grouped by CMV viremia: aviremic, low viremic and high viremic: P=0.7 for aviremic vs CMV level <1000 IU/ml (low viremic), p=0.02 for aviremic vs ≥1000 IU/ml (high viremic), p=0.08 for CMV level <1000 IU/ml (low viremic) vs ≥1000 IU/ml (high viremic).
